# Regulation of Onecut2 by miR-9-5p in Japanese encephalitis virus infected neural stem/progenitor cells

**DOI:** 10.1128/spectrum.03238-23

**Published:** 2024-02-06

**Authors:** Shivangi Sharma, Atreye Majumdar, Anirban Basu

**Affiliations:** 1 National Brain Research Centre, Manesar, Haryana, India; Indian Institute of Science, Bengaluru, Karnataka, India

**Keywords:** NSCs, JEV, miRNA, Onecut2, miR-9-5p, neurosphere

## Abstract

Japanese encephalitis virus (JEV) is one of the major neurotropic viral infections that is known to dysregulate the homeostasis of neural stem/progenitor cells (NSPCs) and depletes the stem cell pool. NSPCs are multipotent stem cell population of the central nervous system (CNS) which are known to play an important role in the repair of the CNS during insults/injury caused by several factors such as ischemia, neurological disorders, CNS infections, and so on. Viruses have evolved to utilize host factors for their own benefit and during JEV infection, host factors, including the non-coding RNAs such as miRNAs, are reported to be affected, thereby cellular processes regulated by the miRNAs exhibit perturbed functionality. Previous studies from our laboratory have demonstrated the role of JEV infection in dysregulating the function of neural stem cells (NSCs) by altering the cell fate and depleting the stem cell pool leading to a decline in stem cell function in CNS repair mechanism post-infection. JEV-induced alteration in miRNA expression in the NSCs is one of the major interest to us. In prior studies, we have observed an altered expression pattern of certain miRNAs following JEV infection. In this study, we have validated the role of JEV infection in NSCs in altering the expression of miR-9-5p, which is a known regulator of neurogenesis in NSCs. Furthermore, we have validated the interaction of this miRNA with its target, Onecut2 (OC2), in primary NSCs utilizing miRNA mimic and inhibitor transfection experiments. Our findings indicate a possible role of JEV mediated dysregulated interaction between miR-9-5p and its putative target OC2 in NSPCs.

**IMPORTANCE:**

MicroRNAs have emerged as key disease pathogenic markers and potential therapeutic targets. In this study, we solidify this concept by studying a key miRNA, miR-9-5p, in Japanese encephalitis virus infection of neural stem/progenitor cells. miRNA target Onecut2 has a possible role in stem cell pool biology. Here, we show a possible mechanistic axis worth investing in neurotropic viral biology.

## INTRODUCTION

Japanese encephalitis virus (JEV) belonging to the *Flaviviridae* family of positive-strand RNA viruses is a major cause of encephalitic endemic in the South East Asia, a few parts of Australia, and the Western Pacific islands (1, 2). In patients who develop the clinical illness, the fatality rate could be as high as 30% and patients among the survivors suffer through long-term neurological sequelae such as frequent seizures, inability to speak, headaches, and loss of motor functions (3, 4). Neural stem cells (NSCs) are pluripotent cells of the brain that have capacity to proliferate and differentiate into neurons and glia. Residing in the sub-ventricular zone (SVZ) and hippocampal dentate gyrus area of the mammalian brain, these NSCs remain active and often respond to the signals provided by the brain injury/insults by migrating, proliferating, and differentiating during traumatic brain injury, ischemia, and in many other neurodegenerative diseases (5–9). These signals include several growth factors and neurotransmitters that are secreted by the injured cells that ultimately affect the neurogenesis (10). NSCs are vulnerable to several neurotropic viral infections. Hence, alterations in their general homeostasis can lead to the dysregulation of the fine tune orchestrated pathways (11–14). In pediatric JEV infection cases, the virus infection of NSC can lead to impaired repair functions leading to motor and cognitive deficits and delayed developmental milestones in survivors (15).

miRNAs are non-coding RNAs that are approximately 20–25 nucleotide long and are known to regulate diverse cellular processes through the assembly of RISC (miRNA-induced silencing complexes) that binds in complementarity to their mRNA targets and subsequently represses their transcription or translation (16, 17). Many studies have suggested a role of certain tissue-specific miRNAs in regulating viral infections (18–21). Neurotropic viruses are reported to hijack the host miRNA machinery present in NSCs leading to dysfunctional cell activity, immature neuronal differentiation, and even cell death (22–24). With the help of miRNA assessment via microarray in human tissues, a previous study from our laboratory has reported the role of miRNAs in JEV infected NSCs, wherein expressions of few miRNAs were found to be consistently downregulated. One such dysregulated miRNA in JEV infected NSPCs was miR-9-5p, which is of primary interest in our study (24). miR-9-5p is a highly conserved miRNA and is a known regulator of neurogenesis (25–27). It is abundantly expressed in the neurogenic regions of the developing and adult nervous system, and many of the developmental processes such as neurogenesis, neuronal cell migration, and proliferation are known to be regulated by this miRNA (28–31). In this study, we have explored the role of miR-9-5p in JEV infected mouse NSCs. With the help of user-friendly miRNA-target interaction platforms such as TargetScan and miRTarBase, Onecut2 (OC2) was identified as a putative target of miR-9-5p. We further checked for its expression in JEV infected NSCs and interestingly, there was an increase in the abundance of OC2 expression levels in the JEV infected samples. This study offers insights into the dysregulated interaction between miR-9-5p and OC2 in NSCs following JEV infection and highlights how viral infection of NSCs can modify the interaction between host miRNA and its mRNA target.

## MATERIALS AND METHODS

### Animals

Both female and male BALB/c mice (purchased from Jackson Laboratory, Bar Harbor, ME) were harbored together along with their respective mothers and were maintained at constant temperature and humidity and under a 12 h light/12 h dark cycle in the animal facility of National Brain Research Centre. Animals were handled with utmost care following recommended animal handling practice guidelines issued by the Committee for the Purpose of Control and Supervision of Experiments on Animals, Ministry of Environment and Forestry, Government of India. All the experiments were performed following approval from the Animal Ethics Committee of National Brain Research Centre (NBRC/IAEC/2018/139).

### Virus propagation

The JEV GP78 strain used in this study was initially isolated from the post-mortem brain tissue of a woman patient in 1978 during an encephalitis outbreak in Gorakhpur, India, and subsequently characterized in 1999 (32). Suckling BALB/c mice of either sex were intracranially infected with JEV GP78 strain and housed with their mothers. All animals developed encephalitic symptoms 72–96 h post-infection (hpi). At this time, brain samples were collected, and 10% of tissue suspension was prepared in minimum essential medium (MEM) followed by centrifugation at 15,000 *g* for 30 min. Virus titer was determined using plaque assay in porcine kidney cells (PS cell). Serial dilution of the virus thus obtained was used to infect the monolayer of the PS cell. At 2 hpi, the cells were washed with phosphate buffer saline (PBS) and an overlay of solution containing 1% low melting agarose, 1× MEM, and fetal bovine serum (FBS) was used to cover the monolayer of infected cells and kept at 37°C in 5% CO_2_ for 96 h to observe the formation of plaques (33).

### Neurosphere culture

SVZ from BALB/c mouse pups (P7) was dissected out aseptically in phosphate buffer with 1 mM MgCl_2_ and 0.6% glucose and minced mechanically. The neurosphere culture was performed as described before (34). The secondary neurospheres were allowed to form for 7–8 days, following which number of spheres and diameter of spheres were determined. All other *in vitro* experiments were carried out after a minimum of two passages and under cell density of 4 × 10^3^ cells/cm^2^ in 12-well plates. Differentiation experiment was performed after seeding the cells in poly-D-lysine (PDL)-coated chamber slides as described before (35).

### Cell culture

Neural stem cell line C17.2 was used for all the *in vitro* neural stem/progenitor cell (NSPC)-related experiments. Cells were maintained in Dulbecco's Modified Eagle Medium (DMEM) containing 10% FBS and 5% horse serum, L-glutamine (2 mM), penicillin (100 units/mL), and streptomycin (100 µg/mL). Human neuroblastoma cell-line SH-SY5Y (a kind gift from Dr. S. Levison, Rutgers University, New Jersey Medical School) was maintained using DMEM/F12 media supplemented with 10% FBS, penicillin (100U/mL) and streptomycin (100 μg/mL). HEK (Human Embryonic Kidney) cell line was used for luciferase assay-related transfection experiments. HEK cells were maintained in low glucose DMEM containing 10% FBS penicillin (100 units/mL) and streptomycin (100 µg/mL). All cells were seeded at the desired density in culture plates as per the requirements for different experiments.

### Viral infection of cells

All cells were seeded at the desired density in culture plates as per the requirements for different experiments. After the cells reached 80% confluence, they were further incubated for 2 h in serum-free medium and infected with JEV at the multiplicity of infection (MOI) of 3. The cells were then gently washed with PBS and maintenance media containing 1% serum were added. Cells were harvested at different times for the time kinetic study.

### Transfection with mimic and inhibitors

Transfection of cells to overexpress or inhibit miR-9-5p was performed with mimic of mouse miR-9-5p (dsRNAs that mimic mature endogenous miR-9-5p) or with miR-9-5p inhibitor (modified ssRNAs that specifically inhibit the endogenous miR-9-5p activity) (Qiagen), respectively, using Lipofectamine-3000 Transfection Reagent (Invitrogen) according to the manufacturer’s instructions. At 24 h and 36 h following transfection with inhibitor and mimic, respectively, the cells were harvested or infected with JEV for the specific times, and then the abundances of the miRNAs, mRNAs, and proteins were analyzed. Negative controls of the mimic or inhibitor (Qiagen) were used in the transfections as the matched controls. An equal volume of Lipofectamine-3000 reagent without any nucleic acid was treated to mock transfection cells.

### Immunocytochemistry

Cells were fixed with 4% paraformaldehyde for 15 min and then gently washed with PBS twice. The fixed cells were then permeabilized with 0.5% PBS with Triton X (PBXT) for 20 min and then were subjected to washes with PBS thrice. The cells were then blocked with 5% bovine serum albumin and 5% goat serum for about an hour. Primary antibody dilution of 1:250 (for OC2) was then added to the respective chambers and cells were incubated for 1 h followed by washes with 1% PBXT (5 times for 5 min each). The cells were then incubated with secondary antibody for 20 min (anti-rabbit Alexa 488 and Alexa 594) followed by gentle washes with 0.1% PBXT 7 times for 5 min each. The slide was then mounted with 4′,6-diamidino-2-phenylindole (DAPI) and observed under fluorescence microscope.

### Immunoblotting

Whole tissue/cell extract was isolated as previously described (36). Utilizing bis-cinchoninic acid calorimetric method, equal concentrations of protein were loaded and were resolved using SDS-PAGE followed by transfer of the nitrocellulose membrane. Membranes were blocked in milk-**T**ris **B**uffer **S**aline in **T**ween-20 (TBST) solution and incubated with the respective primary antibodies OC2 (1:2,000; Bioss), NS3 (1:10,000; Genetex), and β-actin (1:20,000; Sigma-Aldrich), respectively, at 4°C overnight. Next day, following extensive washes with TBST, blots were incubated with **H**orse **R**adish **P**eroxidase (HRP)-labeled secondary anti-rabbit antibodies (1:7,500; Vector Laboratories) for 2 h. Subsequently, after appropriate washes with TBST, blots were then developed using chemiluminescence reagent (Millipore), and images of the respective blot were taken with chemigenius bioimaging system (Uvitech Cambridge). Each blot was normalized with β-actin (after stripping and re-probing with the β-actin antibody). ImageJ was used for the analysis of the blots.

### Polymerasechain reaction

To determine the viral RNA expression, total RNA was isolated from JEV infected C17.2 cells by using TRI Reagent (Sigma-Aldrich), and using Verso cDNA Synthesis Kit (Thermo Fisher Scientific) and miScript cDNA kit (Qiagen), 250 ng of RNA was reverse transcribed. For quantitative determination of mature mRNA and miRNA abundances, quantitative real-time PCR (qRT-PCR) analysis was performed. Isolation of total RNA from the treated cells and mouse brain followed by cDNA synthesis was performed as mentioned above. qRT-PCR analysis of mouse genes was performed using Power SYBR Green PCR Master Mix (Applied Biosystems) along with gene-specific primers (Table 1). The relative abundance of an mRNA of interest was determined by normalization to that of GAPDH mRNA through the 2^−∆∆*Ct*^ method (*C*_*t*_ refers to the threshold value). The isolation of miRNA and cDNA preparation was performed as described earlier (21). The primers of mouse miR-9-5p were used as forward primers in qRT-PCR analysis. The procedure of tissue preparation for miRNA isolation from human brain sections was similar to that of mRNA. The U6 snRNA was used as a normalization control. The thermal cycler real-time PCR (Applied Biosystems) was used for qRT-PCR.

**TABLE 1:** List of primers used

S.no.	Gene	Forward primer	Reverse primer
1	GP78	5′-TTGACAATCATGGCAAAC-3′	5′-CCCAACTTGCGCTGAATAA-3′
2	GAPDH	5’- TCTCCCTCACAATTTCCATCC-3′	5’- GGGTGCAGCGAACTTTATTG-3′
3	SDM-OC2	**5’**ATACTGCAA**ACCAAGA**CATTTGTTAC **3’**	**5’** GTAACAAATG**TCTTGGT**TTGCAGTAT3’
4	WT-OC2	5’GCA**GAGCTC**ACTGGAAAAACTTTCTATTTGTAGTGAGA-3′	5’- GCT**TCTAGA**AGATGCAAGAGACACAGAAAATATCCCTC-3′
5	Luc-OC2	5’ AAGGGCGGCAAGATCGCCGTGTAA3’	

### Plasmid construction and dual-luciferase assay

Freely available software, TargetScan Mouse 8.0, was used to predict candidate target genes of miRNA-9-5p. OC2 was predicted with 14 conserved binding sites for the microRNA. Hence, we went on to clone a segment of OC2 3′ **U**n**t**ranslated **R**egion of messenger RNA (UTR) into pmirGlo reporter plasmid (Promega). A 1,352 bp segment of the 3′ UTR of mouse OC2 (carrying three miR-9-5p binding sites) was amplified by PCR from mouse cell line C17.2 cDNA (Table 1). The DNA fragment was inserted between XbaI and SacI sites in the multiple cloning site downstream of the firefly luciferase gene. Site-directed mutagenesis performed with the SDM primers (forward and reverse; Table 1) generated mutant clones. A PCR-based method involving Phusion High Fidelity DNA Polymerase and DpnI (New England Biolabs) was performed for mutagenesis. The presence of OC2 insert in wild type and mutant plasmids was confirmed using a combination of vector-specific luc-OC2 forward primer and insert-specific WT-OC2 reverse primer. Subsequently, the WT and SDM constructs were confirmed by Sanger sequencing at Regional Centre for Biotechnology, Faridabad, India.

HEK cells were transfected with pmirGLO reporter plasmid containing the wild-type/mutate OC2 3′ UTR along with either miR-9-5p mimic or miR-control using Opti-MEM and Lipofectamine-3000. Cells were maintained in culture for at least 36 h after transfection and then processed using the Dual-Luciferase Reporter Assay System (Promega; catalogue: E4030) according to the manufacturer’s recommendations. Firefly luciferase activity was normalized using renilla luciferase activity encoded by the same pmirGlo reporter plasmid.

### Statistical analysis

All experiments were performed in triplicate unless otherwise indicated. Statistical difference between two groups was analyzed using student’s two-tailed unpaired *t*-test while the comparison involving multiple groups was assessed using one-way analysis of variance (ANOVA) followed by post hoc test. *P* < 0.05 was considered statistically significant, and the results are expressed as mean ± SEM and the statistical analysis was performed in GraphPad Prism 9 (version 9.5.1).

## RESULTS

### miR-9-5p expression is decreased in NSCs during JEV infection *in vivo*

**Fig 1:**
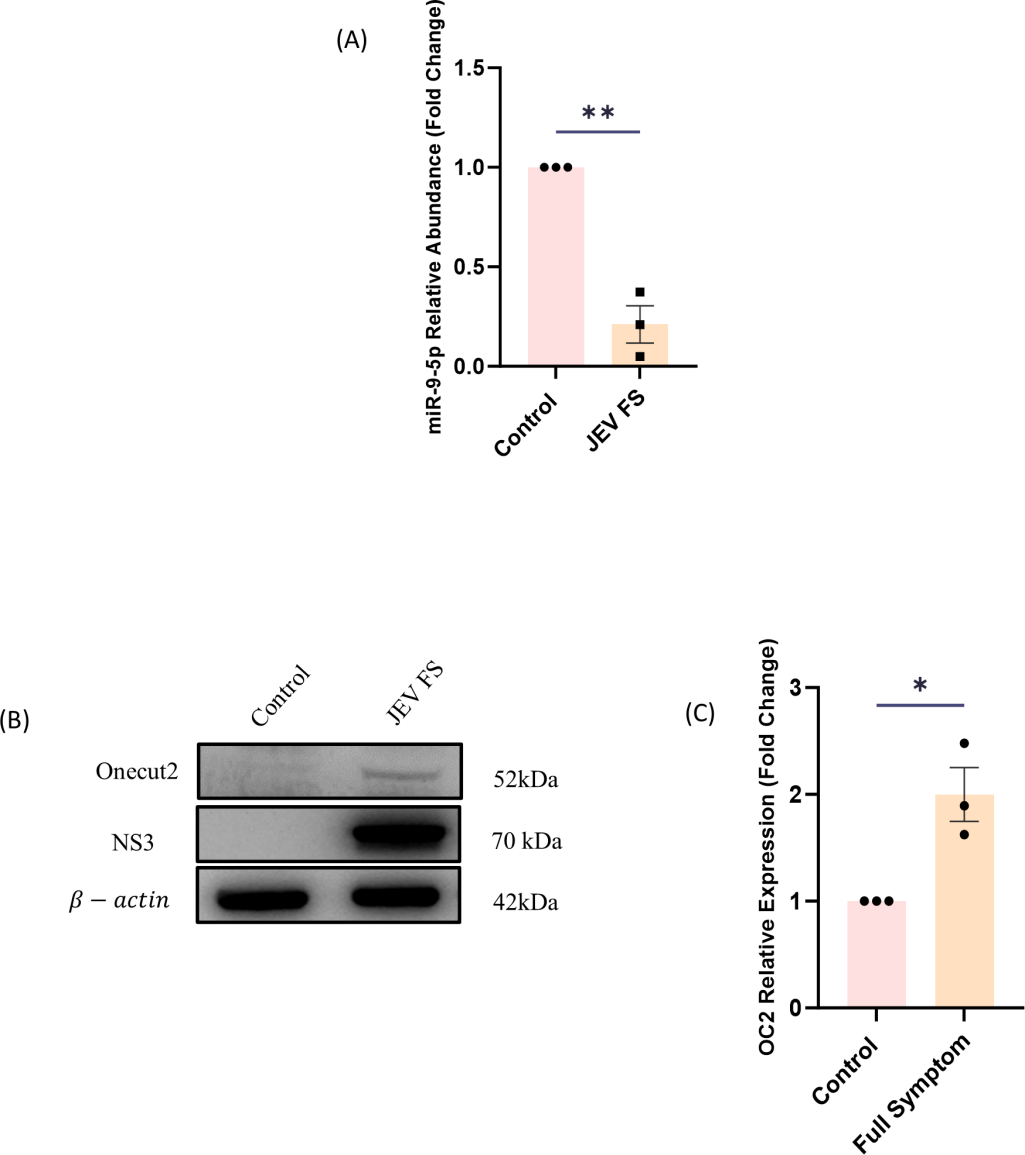
miR-9-5p expression is reduced in JEV infected neural stem cells: BALB/c mouse model was used for *in vivo* JEV experiments. Ten-day-old mice were either mock infected with PBS or infected with JEV (3 × 10^4^ PFU). The animals were then sacrificed after manifestation of JEV full symptom and the SVZ section was aseptically dissected out. The relative abundance of miR-9-5p to that of U6 was measured through qRT-PCR ( Fig.1A) and protein lysate was analyzed through immunoblot assay to measure the expression of OC2 (Fig. 1B and C) in infected animal samples (significance was calculated using two-tailed t-tailed *t*-test; ***P = 0.0002).

We sought to understand the link between miR-9-5p expression and JEV infection in mouse NSPCs. BALB/c mice at post-natal age 10 (P10) were infected with JEV and were sacrificed upon manifestation of JEV full symptom following which the SVZ brain section was collected aseptically. The SVZ brain section was subjected to qRT-PCR and immunoblotting to check for the expression levels of miR-9-5p post JEV infection in NSPCs. qRT-PCR analysis revealed that there is a decreased expression of miR-9-5p in NSPCs following JEV infection (Fig. 1A). To gain more insights into the mechanism underlying miR-9-5p function in our model of JEV infection, we analyzed probable target genes of miR-9-5p with the help of widely used and freely available miRNA target prediction database TargetScan and found that OC2 had the highest interaction score. Immunoblotting assay of SVZ lysate revealed a significantly increased expression of the OC2 levels in JEV infected NSPCs in comparison to mock infected (control) animals (Fig. 1B and C).

### miR-9-5p expression is decreased in NSCs during JEV infection *in vitro*

**Fig 2:**
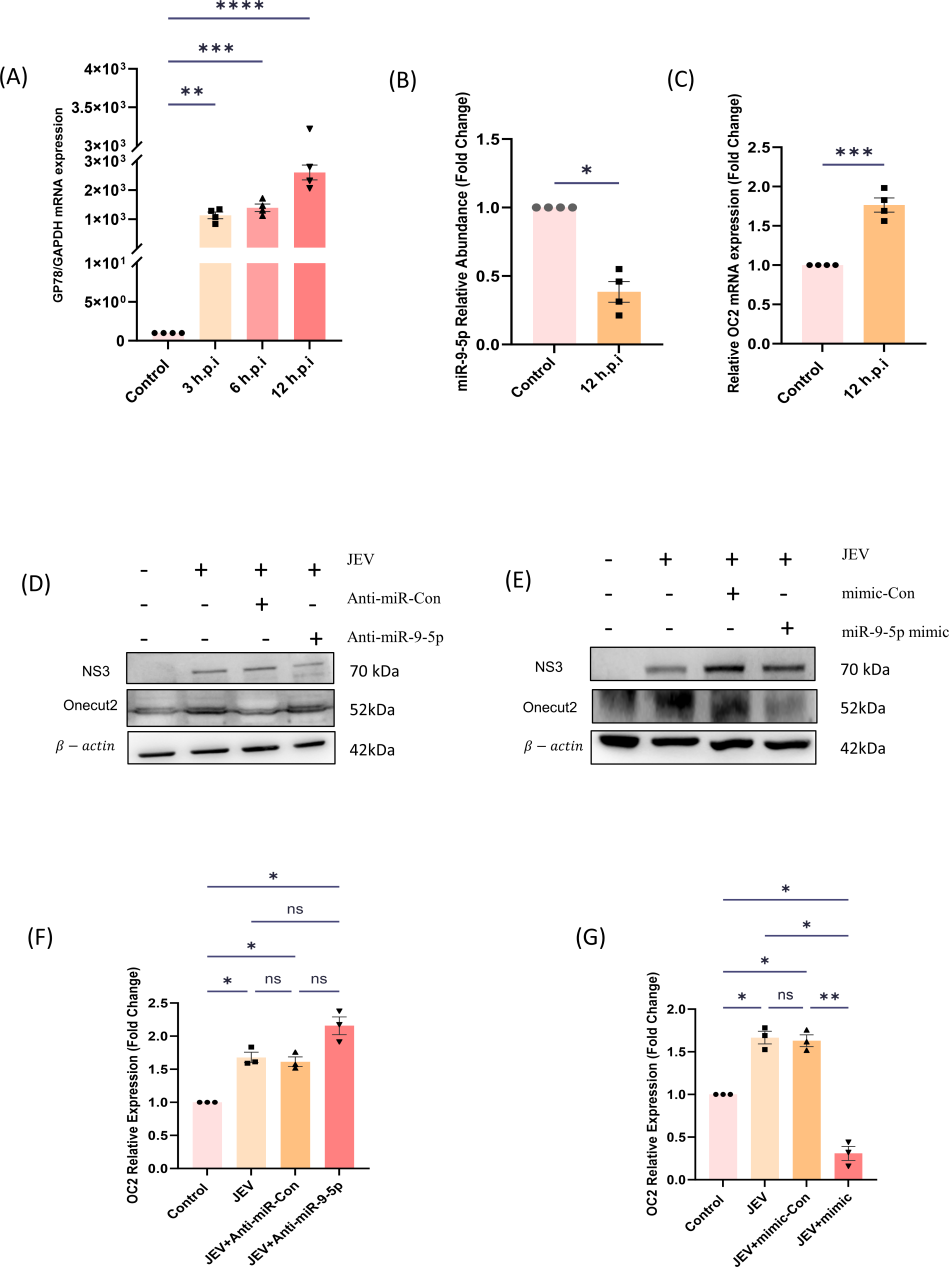
miR-9-5p expression is reduced in JEV infected neural stem cells: (A) C17.2 cells were left uninfected (Control) or were infected with JEV at 3 MOI (multiplicity of infection) at the indicated time points before the abundance was measured by qRT-PCR analysis and normalized to that of GAPDH mRNA (the data here are represented as bar chart with mean ± SEM where significance was calculated using one-way ANOVA. **P < 0.0021; ***P < 0.0002; ****P < 0.0001, indicating significant post hoc comparison, minimum of three biological replicates). (B and C) C17.2 cells were infected at 3 MOI and the samples were collected at 12 hpi to measure the relative abundance of miR-9-5p to that of U6 through qRT-PCR (significance was calculated using two-tailed t-tailed *t*-test; *P < 0.0286); while the OC2 expression was measured after normalizing with GAPDH mRNA (significance was calculated using two-tailed t-tailed *t*-test; ***P < 0.0001) compared to uninfected cells. (D through G) C17.2 cells were transfected with either miR-9-5p mimic and inhibitor to measure the expression levels of OC2 through Western blot (the data here are represented as bar chart with mean ± SEM where significance was calculated using one-way ANOVA. *P < 0.0132; ***P < 0.0001, indicating significant post hoc comparison, minimum of three biological replicates).

**Fig 3:**
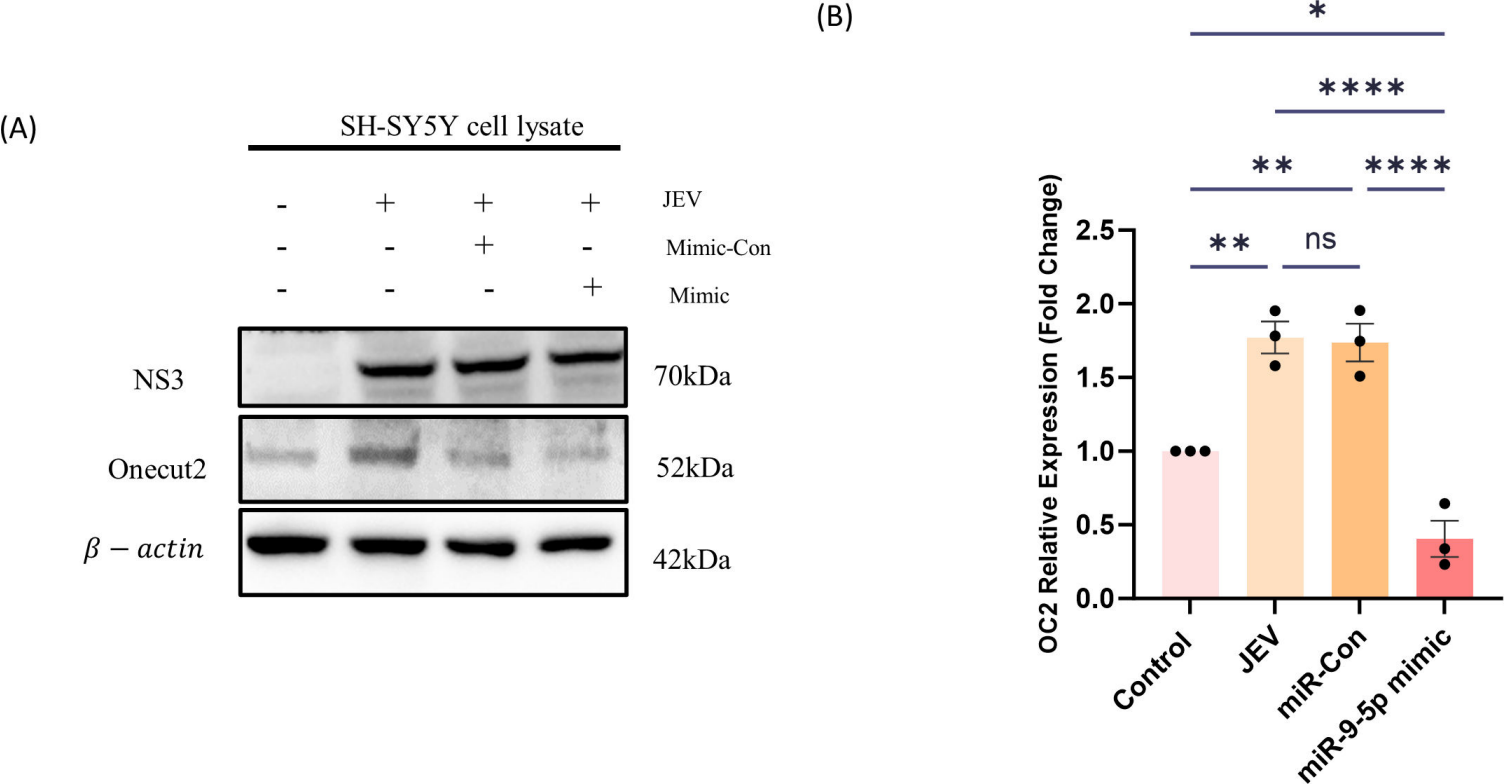
miR-9-5p expression is reduced in JEV infected SH-SY5Y cells: SH-SY5Y cells were transfected with miR-9-5p mimic and mimic control along with JEV infection to measure the expression levels of OC2 through Western blot (the data here are represented as bar chart with mean ± SEM where significance was calculated using one-way ANOVA. *P < 0.0132; ***P < 0.0001, indicating significant post hoc comparison, minimum of three biological replicates).

To validate our *in vivo* findings, in our *in vitro* model of JEV infected NSPCs, time kinetic assays of JEV infection were performed to observe at which time point the expression of miR-9-5p was downregulated the most (Fig. 2A and B). At 12 hpi, the abundance of miR-9-5p was markedly reduced in JEV infected NSCs compared in comparison to uninfected ones. In parallel, the relative expression of OC2 was inversely proportional to the miR-9-5p expression (Fig. 2C). Further, transfection with either miR-9-5p mimic or inhibitor confirmed that, upon increased miR-9-5p expression, OC2 abundance is reduced or vice versa in comparison to their respective controls (Fig. 2D through G). Further, we validated our *in vitro* findings of JEV infection in NSPCs in the neuroblastoma cell line SH-SY5Y, wherein the immunoblot assay revealed that, upon transfection with miR-9-5p mimic and further infection with JEV (12 hpi), the OC2 expression levels were found to be decreased in mimic + infected samples w.r.t. JEV only or Scr + JEV samples (Fig. 3A and B).

### miR-9-5p expression is reduced in JEV infected neurosphere

**Fig 4:**
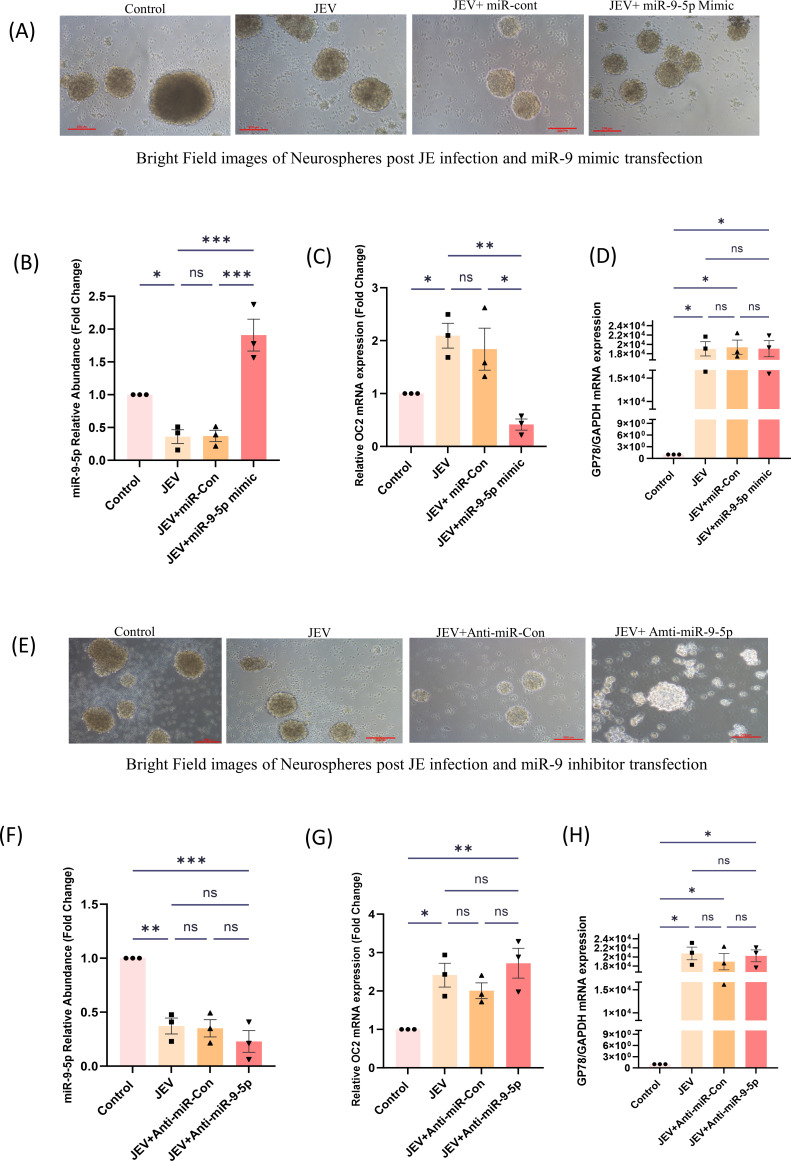
Relationship between miR-9-5p and OC2 in JEV infected primary NSCs: (A and E) Bright field images of the mock (Control) or JEV infected neurospheres transfected with either miR-9-5p mimic/mimic control (miR-Con) or miR-9-5p inhibitor (Anti-miR-9–5p) or inhibitor control (Anti-miR-Con). Scale bar represents 100 µm (20 × magnification). (B and C; **F and G**)The relative abundance of miR-9-5p to that of U6 and OC2 to that of GAPDH was measured through qRT-PCR (the data here are represented as bar chart with mean ± SEM where significance was calculated using one-way ANOVA. *P < 0.0335; **P < 0.0064, indicating significant post hoc comparison, minimum of three biological replicates).

**Fig 5:**
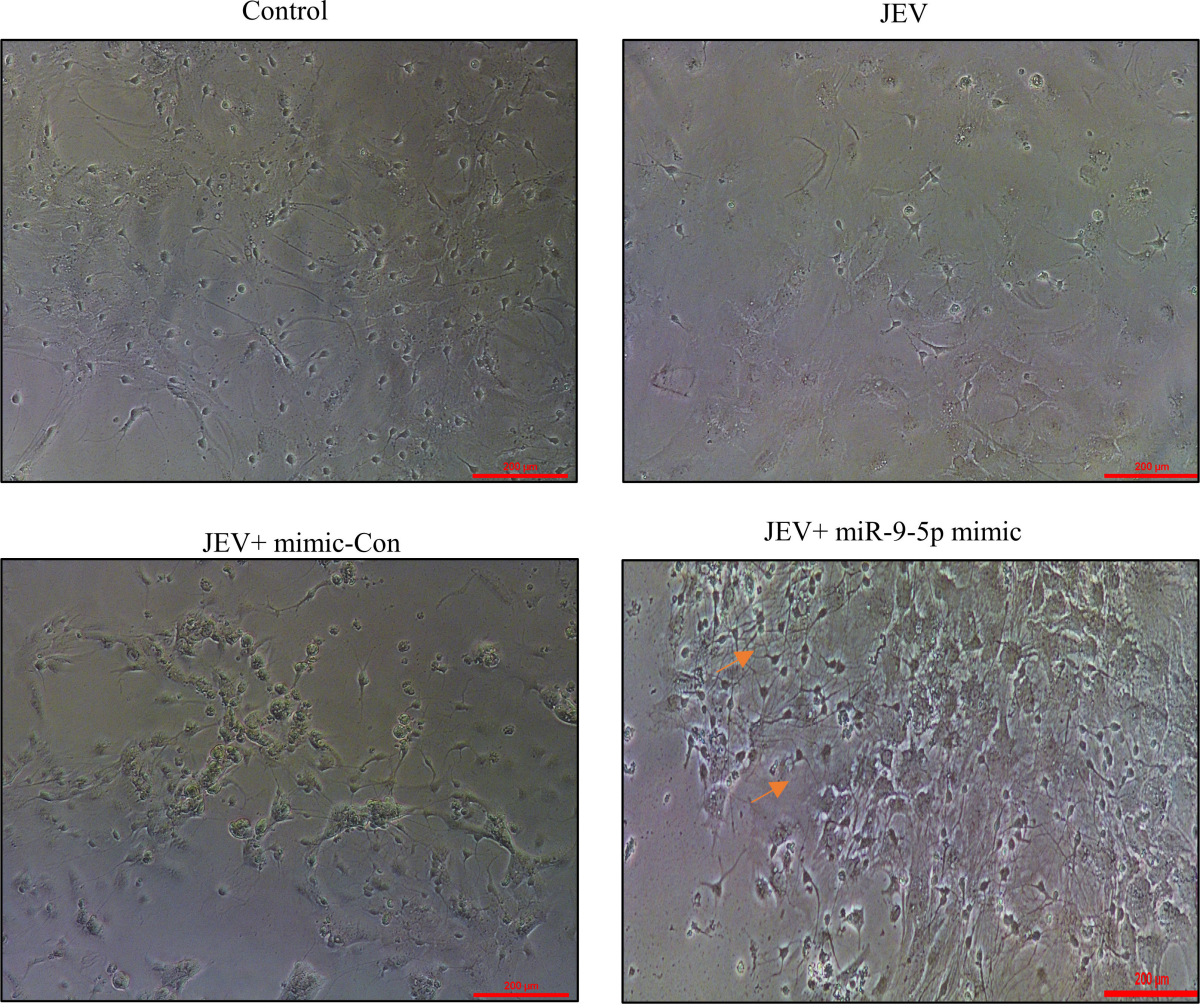
miR-9-5p improved neuron formation in JEV infected differentiating primary NSCs: Bright field images of neurosphere subjected to differentiation post transfection (with mir-9-5p mimic) and JEV infection. Scale bar denoted in each micrograph measures 100 µm (20 × magnification).

To further validate our *in vitro* and *in vivo* findings of NSC infection by JEV, primary neurosphere culture was utilized. The primary neurospheres were cultured for 3 weeks and were then seeded for transfection and JEV infection experiments with mimic or inhibitors (anti-miR), respectively (bright field images; Fig. 4A and E). The relative abundance of the miR-9-5p to that of U6 snRNA was then measured through qRT-PCR. In the neurospheres transfected with miR-9-5p mimic, the relative levels of miR-9-5p were found to be upregulated in the mimic transfected and JEV infected samples in comparison to only JEV infected ones. Subsequently, its target expression (OC2) was found to be lower in the transfected and infected (mimic + JEV) samples in comparison to the only infected ones (Fig. 4B through D). Opposing results were observed upon inhibiting the miR-9-5p expression that resulted in increasing the OC2 abundance however non-significant in comparison to the only infected ones (Fig. 4F through H). Neurospheres (transfected with mimic and infected with 3 MOI of JEV) were also subjected to the differentiation experiments, wherein after transfection with miR-9-5p mimic and infection with JEV, neurospheres were grown in differentiation media in PDL-coated chamber slides for 10 days. Differentiated cells (transfected with mimic and JEV infected) exhibited more neuronal phenotype in bright field images in comparison to the transfected and infected control (Fig. 5).

### miR-9-5p regulates OC2 expression in NSCs

**Fig 6:**
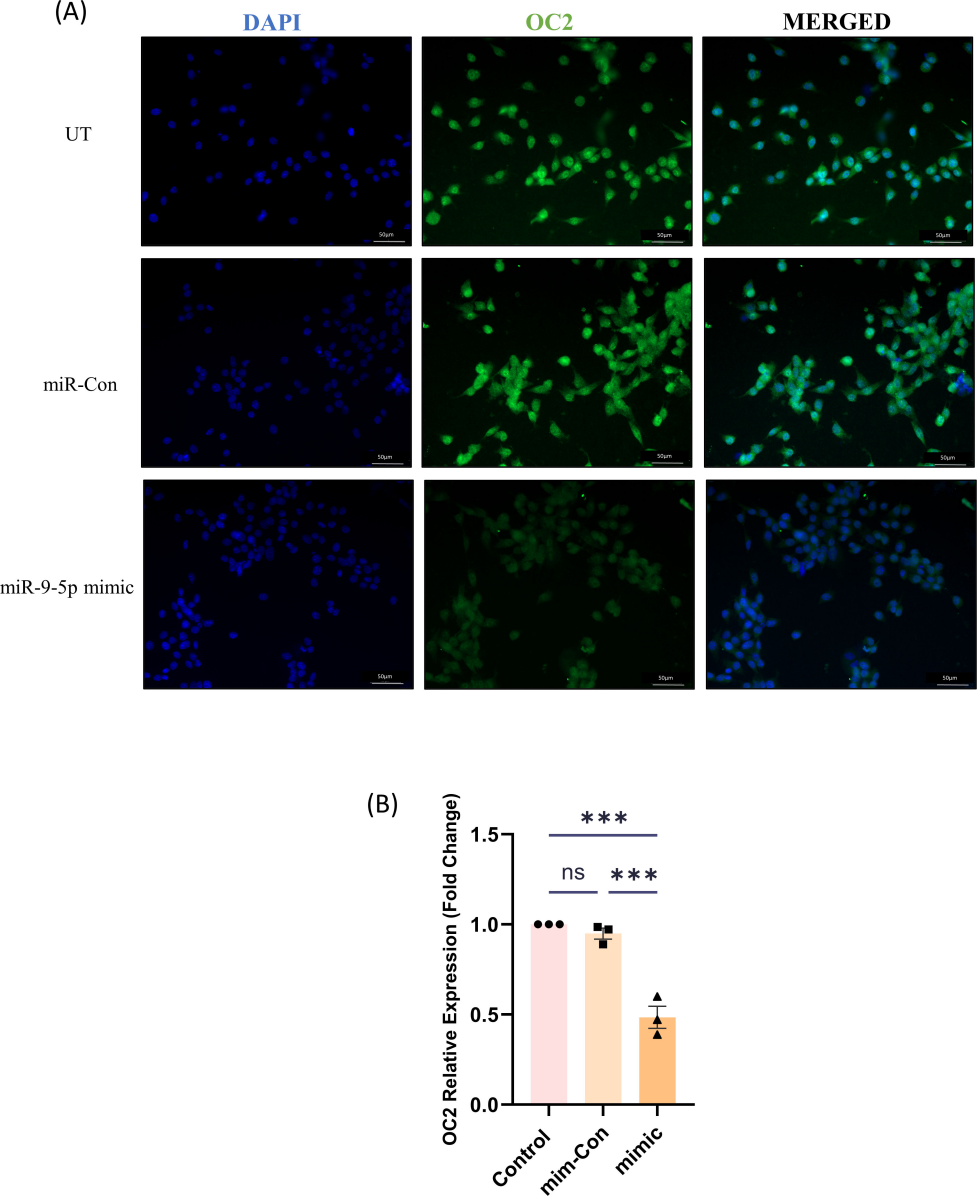
OC2 abundance is regulated by miR-9-5p: Immunocytochemical assay of OC2 expression upon transfection with either miR-control or miR-9-5p mimic in C17.2 cell line. Scale bar denoted in each micrograph measures 50 µm (20 × magnification). Quantitative analysis of OC2 abundance in un-transfected and transfected cells (the data here are represented as bar chart with mean ± SEM where significance was calculated using one-way ANOVA. **P < 0.0002, indicating significant post hoc comparison, minimum of three biological replicates).

To validate that the expression of OC2 is regulated miR-9-5p in NSCs, C17.2 cell line was transfected with either mimic or miR-control and cells were further subjected to the immunocytochemical assay (Fig. 6A). A decrease in OC2 abundance post transfection with mimic was observed, which validated that the abundance of OC2 expression decreases when compared to the miR-mimic control (Fig. 6B).

### miR-9-5p regulates OC2 expression by inhibiting its 3′ UTR region

**Fig 7:**
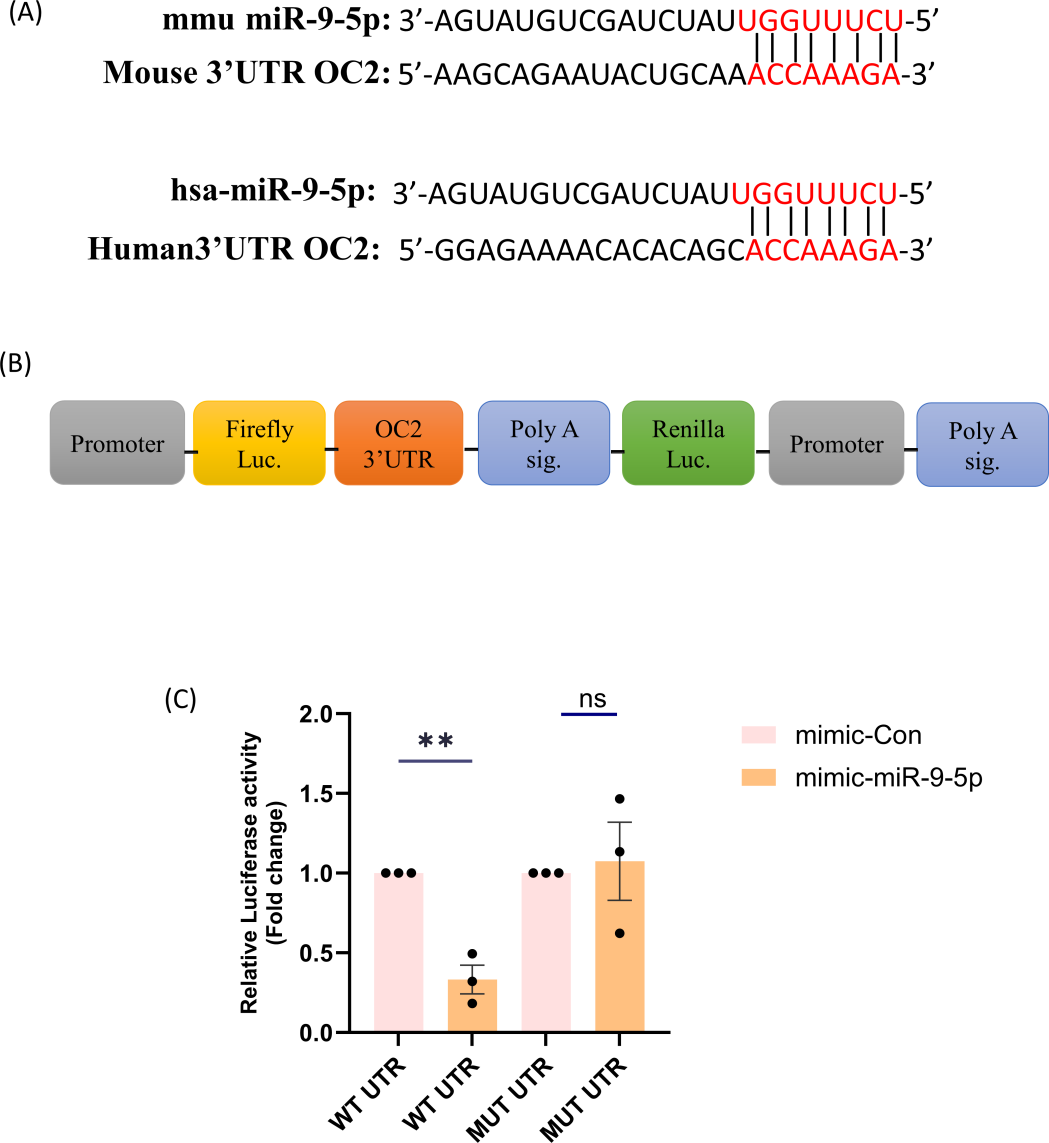
OC2 is a target of miR-9-5p: (**A**) Predicted miR-9-5p binding sites in the 3′ UTR of OC2. (**B**) Diagrams of constructs containing the 3′ UTR of OC2 downstream of a luciferase reporter. The WT 3′ UTR (WT UTR) contains an intrinsic miR-9-5p binding site, whereas the mutant 3′ UTR (Mut UTR) of OC2 contains mutations that eliminated the seed match with miR-9-5p. (**C**) Dual-luciferase assays of HEK cells co-transfected with firefly luciferase constructs containing the WT or mutant 3′ UTRs of OC2 and either the miR-9-5p mimic (Mimic) or the control mimic (Con-Mimic) were performed. Firefly luciferase activity was normalized to renilla luciferase activity. Data are shown as the relative luciferase activity of cells transfected with the miR-9-5p mimic compared to that of cells transfected with the control mimic (the data here are represented as bar chart with mean ± SEM where significance was calculated using two-tailed unpaired *t*-test; **P < 0.0033).

To further verify our findings of miR-9-5p interaction with OC2, we cloned the 3′ UTRs of mouse OC2 into a firefly luciferase reporter vector. We then used mutations in the predicted seed matching site in the 3′ UTRs of OC2 (to test the miRNA-target interactions). HEK cells were then transfected with individual reporters containing wild-type (WT) or mutant (Mut) UTRs together with an miR-9-5p mimic or a mimic control. The miR-9-5p mimic effectively reduced the luciferase activity of the WT UTR reporter compared to that in cells transfected with the mimic control. In contrast, the miR-9-5p-dependent reduction in luciferase activity was disrupted by mutating the 3′ UTR binding sites in OC2. These results suggest that miR-9-5p can regulate the expression of OC2 by binding to its 3′ UTR (Fig. 7A through C).

## DISCUSSION

NSCs are known to play an important role in the central nervous system (CNS) repair during several neurodegenerative disorders and pathological insults leading to their increased proliferation, migration, and differentiation. NSCs are a promising modality in several diseases associated with the CNS as they are known to be able to participate in repair mechanisms (37, 38). To underline the direct relationship between neurological sequelae and viral infections is a challenge since an interplay of several environmental factors and host factors occurs during neurotropic infections (39). One such host factors are miRNAs. These small non-coding RNAs have an important role in fine tuning the gene expression in NSCs during neurotropic viral infections (40–42). Previous reports by our group have established how NSC fate determination and self-renewal capacity are abrogated during JEV infection and how abundance of certain host miRNAs was getting affected post JEV infection in NSCs (24, 35). An interesting outcome among these reports was the reduction in the miR-9-5p levels in JEV infected NSCs. As previously stated, miR-9-5p is a known regulator of neurogenesis of NSCs that are enriched in the neurogenic areas of the CNS (26, 43, 44). One study has depicted the role of miR-9-5p in the suppression of astrogliogenesis by targeting a transcription factor, PTBP1 (an activator and transducer of JAK-STAT pathway), resulting in the inhibition of STAT phosphorylation and inhibiting astrogliogenesis (31). However, its function in the JEV infected NSCs is yet to be fully explored. In our present study, we establish that the expression of miR-9-5p is inhibited during the JEV infection in murine model of NSCs (Fig. 1). Widely annotated miRNA target interaction platform, TargetScan, revealed OC2 as a significant target of miR-9-5p. OC2 is an important transcription factor expressed in the CNS and is reported to be required during the development of the CNS regulating the differentiation of the NSCs. Also, in another interesting study by van der Raadt *et al.* (45), it was reported that overexpression of OC2 can lead to the expression of genes to non-neuronal pathways such as apoptotic mitochondrial changes and type-I interferon signaling. These pathways are critically studied by our group and are reported to play an important role post JEV infection (45–49).

Our *in vivo* qRT-PCR data obtained from the RNA isolated from the SVZ section of JEV infected animals revealed a decrease in miR-9-5p expression. Subsequently, inhibition of miR-9-5p resulted in elevated OC2 (its mRNA target) expression during JEV infection. This was observed in the immunoblots of the cell lysates isolated from SVZ of infected animals in comparison to control (mock infected) animals. Through the *in vitro* validation study utilizing mimic and inhibitor transfection experiments along with JEV infection, we were able to validate OC2 as a mRNA target of miR-9-5p during this *Flaviviral* infection of NSCs as well as in SH-SY5Y cell line. Transfection experiments with mimic and inhibitor in *ex vivo* culture of primary NSCs (neurosphere culture) also exhibited similar results to that of our *in vitro* findings. Furthermore, immunocytochemistry experiments after transfection with miR-9-5p mimic also revealed that, upon transfection with the miR-9-5p mimic, OC2 expression levels were drastically reduced in comparison to its transfection control. Additionally, to establish that the repression of OC2 expression is regulated by miR-9-5p, dual transfection with 3′ UTR of OC2 transcript (WT UTR) and mimic was performed. When subjected to the dual-luciferase assay with miR-9-5p mimic transfection, the luciferase activity was abrogated, when compared to the transfection control. On contrary, upon mutating the miRNA binding site in the 3′ UTR of OC2 transcript (MUT UTR) through the site-directed mutagenesis, there were no significant changes in the luciferase activity when compared to the respective transfection control. Collectively, these observations demonstrate the interaction between OC2 and miR-9-5p in NSC model and its implications in JEV infection. In 2017, Madelaine *et al*. in their study have also reported a similar interaction of miR-9-5p with OC2 in the NSCs, further supporting our observations (44). As previously stated in the study of our laboratory where miR-9-5p levels found to be downregulated post JEV infection in hNSCs, IL-6 was also found to be a validated target of this miRNA in JEV infected hNSCs. In a separate study, IL-6 expression was found to be elevated in JEV infected NSCs isolated from the *in vivo* murine model of JEV infection in comparison to the mock infected ones (50). IL-6 besides its pro-inflammatory nature has another role to play in the CNS as it is known to play an important role as a neurotrophic and differentiation factor in the CNS (51). Post-natal increased circulation of IL-6 is also known to acutely increase proliferation of NSCs but ultimately depletes the NSC pool (52). All of these evidence points toward an important role of miRNA-9-5p in maintaining the homeostasis NSCs that get abrogated post JEV infection. However, more insights into the mechanism of action of miR-9-5p mediated neurogenesis in NSC model of JEV infection needs to be carefully examined. Additionally, miRNA-mRNA interactions in regulating the molecular processes that affect the expression of the mRNA target genes are not the only sole processes, and regulation of feedback and feedforward cellular fates are taken care of by the interplay of many other molecular networks that are at play. To the best of our knowledge, this study is the first to examine the relationship of miR-9-5p interaction with its target OC2 in JEV infected NSCs.
